# Price-Minimizing Behaviors in a Cohort of Smokers before and after a Cigarette Tax Increase

**DOI:** 10.3390/ijerph13060608

**Published:** 2016-06-17

**Authors:** Anne Betzner, Raymond G. Boyle, Ann W. St. Claire

**Affiliations:** 1 Professional Data Analysts, Inc., Minneapolis, MN 55414, USA; abetzner@pdastats.com; 2 ClearWay Minnesota, Minneapolis, MN 55425, USA; astclaire@clearwaymn.org

**Keywords:** cigarette tax, price minimizing behaviors, cohort, in-depth interviews

## Abstract

Cigarette tax increases result in a reduced demand for cigarettes and increased efforts by smokers to reduce their cost of smoking. Less is known about how smokers think about their expenditures for cigarettes and the possible mechanisms that underlie price-minimizing behaviors. In-depth longitudinal interviews were conducted with Minnesota smokers to explore the factors that influence smokers’ decisions one month prior to a $1.75 cigarette tax increase and again one and three months after the increase. A total of 42 were sampled with 35 completed interviews at all three time points, resulting in 106 interviews across all participants at all time points. A qualitative descriptive approach examined smoking and buying habits, as well as reasons behind these decisions. A hierarchy of ways to save money on cigarettes included saving the most money by changing to roll your own pipe tobacco, changing to a cheaper brand, cutting down or quitting, changing to cigarillos, and buying online. Using coupons, shopping around, buying by the carton, changing the style of cigarette, and stocking up prior to the tax increase were described as less effective. Five factors emerged as impacting smokers’ efforts to save money on cigarettes after the tax: brand loyalty, frugality, addiction, stress, and acclimation.

## 1. Introduction

Raising tobacco taxes is considered the single most important step governments can take to reduce the consumption of tobacco products [1]. Higher tobacco taxes are an important public health policy goal because higher prices lead to multiple positive effects including less initiation of smoking by young people [2], less participation in smoking as some smokers stop smoking [2,3], and lower consumption as some smokers reduce how much and how often they smoke [2,4].

Although higher prices motivate some to quit, it is common for other smokers to find ways to reduce their cost of smoking and thus minimize the effect of a cigarette price increase [5]. There is an established body of literature that has detailed the price-minimizing behaviors of smokers including buying cheaper brands [3,6,7], buying cigarettes from low-tax or untaxed sources [7,8], using loose tobacco to roll their own cigarettes [7,9,10], using coupons and other discounts [4,11], buying different quantities to save money [5,7,10,12], and finding less expensive places to buy cigarettes [3,12]. A much smaller body of literature explores reducing the number of cigarettes smoked to save money [4,9].

The influence of taxes on price-minimizing behaviors is largely based on cross-sectional population-based survey data [4,7,13]. Other types of studies such as longitudinal cohort studies have been limited to exploring the incidence of specific price-minimizing behaviors such as discount cigarette use [14] and brand switching [11], as well as the impact of price-minimizing behavior on subsequent smoking behavior [10]. Despite a call for qualitative exploration of price-minimizing behavior [6], the authors are aware of only one such study, which focuses on low-income smokers’ experiences of financial deprivation and smoking [9]. The article brings to light previously unexplored price-minimizing behaviors, such as finding and smoking butts, and suggests price-minimizing behaviors may be sporadically used as a way to get by when money is tight [9]. However, a fuller exploration of the possible mechanisms that underlie smokers’ efforts to save money may be helpful to guide future policy and price-minimizing research.

The current research seeks to understand from smokers themselves how they think about their expenditures for cigarettes, how their efforts to save money impact their tobacco use, and the possible mechanisms that underlie their efforts to save money. We had an opportunity to explore price-minimizing behaviors in detail and over time with a cohort of smokers who were interviewed before and after a statewide cigarette tax increase. The primary aim of the study was to explore the context and factors that influence smokers’ decisions to minimize the cost of their smoking when faced with a tax-related price increase.

## 2. Experimental Section

In-depth longitudinal repeated interviews were conducted with Minnesota smokers both before and after the State of Minnesota increased the combined cigarette excise and sales tax by $1.75 on 1 July 2013, from $1.60 to $3.35 [15]. At the time, this was the largest U.S. state increase in cigarette taxes since 2000 [15]. Baseline interviews were conducted in June 2013, one month prior to the tax implementation date. Participants were interviewed twice thereafter: one and three months after the tax increase, in August and October 2013, respectively.

### 2.1. Recruitment

Participants were recruited by a national marketing research firm from an existing database of 75,000 Minnesota adults that reflect state residents on key demographic characteristics. Respondents were assessed via telephone on eligibility criteria that included being a current smoker living in Minnesota and consenting to participate in a set of three interviews over four months. A stratified purposeful sample of adults was recruited from eligible participants so that half the sample included women, those with less than a college degree, and residents of the Minneapolis/St. Paul metropolitan area.

### 2.2. Data Collection

A total of 42 adults were sampled and 38 interviews were completed at baseline. A total of 37 and 35 interviews were conducted at a one-month and three-month follow-up, respectively. Over half of the interviews were conducted by the first author, who trained two study contributors (Mao Thao (MT) and Lindsay Larsen (LL)) to conduct the remaining interviews. Interviewers met weekly to discuss the interview protocol and to maintain consistent interviewing across time. One participant who only completed an interview at baseline was excluded from analysis because no follow-up data were available. Another participant who completed interviews at all three time points was excluded because his comments were highly inconsistent across time; interview data at Time 3 suggest that the participant’s responses may have been deliberately untruthful during the first two interviews, perhaps due to being under court supervision for prior illegal activity. Interviews lasted between 30 and 45 min.

Smokers were asked about the type, brand, and style of tobacco they smoked, their smoking and buying habits, and the reasons behind their decisions. Questions on household income, health-related quality of life and perceived stress were also asked. The exploratory and qualitative nature of the study allowed interviewers to probe participant responses to better learn about behaviors to reduce cigarette costs, the reasons for those behaviors, and the context surrounding the behaviors. Table 1 outlines key items in the interview protocol. A $35 incentive was provided after participants completed each interview.

### 2.3. Analysis

With verbal consent, each interview was audio recorded and transcribed and reviewed for accuracy. Four sets of analyses were conducted that encompass a mixed methods approach [16,17]. The overarching analytic strategy was a fundamental qualitative descriptive approach, with the goal of conducting a low-inference analysis leading to a straight-forward presentation of participants’ perceptions and behaviors in everyday language [18]. Data were coded according to methods outlined by Saldana [17].

Using the descriptive questions identified by Saldana [17], the first analysis sought to identify and code price-minimizing behaviors with the goal of describing those behaviors cross-sectionally (at each time point). Final codes for price-minimizing behavior were set after the Time 2 interviews were completed and were based on a total of five readings of the Time 1 and 2 transcripts across staff. Price minimizing behaviors were defined as cigarette-buying and smoking actions that participants reported engaging in for the purpose of saving money. Behaviors were also identified as being currently practiced or having occurred in the past, and if they were new since the last interview (for post-tax interviews at Time 2 and 3). To ensure consistent coding across staff, the study author (Anne Betzner) and other interviewers (MT, LL) independently coded three interviews and compared and resolved differences. All other interviews were coded by one person only. Price-minimizing behaviors were coded in RQDA, a qualitative data analysis program that runs in the statistical programming system R (R Foundation, Vienna, Austria).

The second set of analyses were designed to understand contextual factors associated with price-minimizing behaviors. Due to the large amount of qualitative data, codes were developed by the study author after three complete readings of the full set of interviews based on Saldana’s framing questions [17]. Seven codes were initially identified by the lead study author. After discussion among the study interviewers (MT, LL) codes were consolidated and revised to the five. The consistency of coding is supported by the multiple, careful readings of the interviews. The study author also developed a narrative summary for each code that included quotes from each participant’s three interviews that exemplified the factor.

The third analysis was of quantitative participant demographic characteristics, health-related quality of life, and perceived stress. Data were collated in an Excel database and analyzed using descriptive statistics.

Finally, in order to examine relationships and trends within and across participants over time, data on price-minimizing behaviors, contexual factors associated with these behaviors, and demographic characteristics were analyzed together using Saldana’s analytic and interpretive questions [17]. Based on data from the first three analyses and additional readings of interview transcripts, the lead study author developed a narrative summary for each participant that described his or her trajectory of price-minimizing behaviors and factors associated with these across the three interviews conducted, along with quantitative demographic, health-related quality of life, and perceived stress data. After each participant-specific trajectory narrative was completed, the study author examined findings across respondents to gain a stronger understanding of factors influencing price minimizing behavior. These are five factors associated with price minimizing behavior presented in this study.

The consistency of interpretation in this phase of the analysis is solely dependent on the study author’s multiple readings of the text, and in-depth understanding of data provided. Because participant-specific narratives were used as a data source, participant behavior trajectories or “stories” became evidence of themes described in this paper. These trajectories span multiple time points and reflect lengthy conversations, often resulting in cumbersome quotations; therefore, author-developed narrative summaries of behavior trajectors are provided as evidence in the text of this manuscript. Trajectories were selected for inclusion in this paper because they exemplify the themes identified. Additionally, quotes are included as evidence as applicable.

The Minnesota Department of Health IRB reviewed and approved study protocols, IRB identification number 13-306.

## 3. Results

The analysis was based on 106 interviews from 34 individuals who completed three interviews and two participants who completed two interviews. Approximately half of the 36 participants (53%) were female and their average age was 43.5 years. Participants were about evenly split between living in the Minneapolis/St. Paul metropolitan area (58%) and living outside the metro area (42%). A high school diploma was the highest educational level achieved by 47% of participants and 55% reported an annual household income of less than $35,000. About 14% had an annual household income of less than $15,000, and 25% had an income above $50,000. All participants were current smokers. Most smoked every day; only three of 36 smoked only some days at baseline. The average number of cigarettes per day smoked at baseline was 13.5. Most (75%) smoked 14 cigarettes per day or fewer at baseline; 14% smoked 15 to 24 and 11% smoked 25 or more.

### 3.1. Price-Minimizing Behaviors

At baseline, all 36 participants reported engaging in at least one behavior to reduce the cost of cigarettes at some time in their past, considering past and current price-minimizing behaviors reported at Time 1. The use of coupons was most common (*n* = 28, 78%), followed by shopping around by price (*n* = 25, 69%), using roll-your-own cigarettes or pipe tobacco (*n* = 21, 58%), and changing to cheaper brands (*n* = 20, 56%). Substantial numbers of respondents also reported smoking half and saving it for later (*n* = 17, 47%), cutting down (*n* = 16, 44%), buying by the carton (*n* = 10, 28%), and having changed or tried an e-cigarette to save money (*n* = 10, 28%). Few participants reported changing to cigars or cigarillos (*n* = 4, 11%), stocking up pre-tax (*n* = 4, 11%), sharing fewer cigarettes with their friends (*n* = 3, 8%), buying online (*n* = 2, 6%), buying a pack with someone else (*n* = 2, 6%), or changing to another type of tobacco such as snuff or snus (*n* = 1).

When interviewed at each time point, participants were asked to talk about their efforts to save money, the extent to which they worked in the short and long term, and what participants thought of the strategies more generally. Based on a consideration of the cost savings of each strategy and the contextual factors of how it was typically used, price-minimizing behaviors could be considered as a hierarchy of effective ways to save money at the time they used them and over time. Participants described saving the most money per use by changing to pipe tobacco and using it to make roll-your-own cigarettes, changing to a cheaper brand, cutting down or quitting, changing to cigarillos, and buying online. A majority of participants (26 of 36, 72%) had tried at least one of these strategies at some time during their recent smoking history, and 17 of 34 (50%) were actively using at least one at Time 3. Although using roll-your-own pipe tobacco was common, most reported either not liking the taste of this tobacco or finding it inconvenient, even as a means to save money. Far fewer participants (*n* = 6 of 36, 17%) reported using roll-your-own tobacco for a sustained amount of time during the study period.

Participants’ descriptions of their smoking and cost savings suggested the following were less effective over time to save money: using coupons, shopping around, buying by the carton, changing the style of cigarette (e.g., to 100 s for greater value), and stocking up prior to the tax increase. Couponing and shopping around were the two most common price-minimizing strategies reported, and almost all participants, 35 of 36, had ever used at least one less effective price minimizing strategy. These strategies tended to focus less on changing smoking behavior.

Based on participant reports, a general consensus did not emerge regarding the effectiveness of the following strategies to save money: using in-store specials or promotions, smoking half and saving half for later, changing to or trying e-cigarettes to reduce cost, sharing fewer cigarettes with friends, and buying a pack with someone else. These strategies were about as commonly employed as the more effective ones, with 25 of 36 participants (69%) reporting having ever used one or more.

### 3.2. Changes in Tobacco-Related Behaviors Post-Tax

The number of cigarettes smoked changed for many after the tax. A total of 11 of 34 respondents reduced their use from Time 1–3 (32%), and an additional four reduced their use at Time 2 but did not sustain the reduction at Time 3 (*n* = 15 total, 44%). A total of seven respondents (21%) increased their use at Time 3 with no reduction. A minority of respondents, one-third (*n* = 12), reported no change in the amount of tobacco that they smoked from Time 1–3.

Similarly, price-minimizing behaviors changed post-tax. Almost all participants, 29 of 36 (80%), changed the number of price-minimizing behaviors post-tax. From Time 1–3, many participants (*n* = 16, 55% of 29) reported more price-minimizing behaviors. For others (*n* = 6, 21% of 29) there was a short-term increase that was not sustained. Finally, six of 29 people (21%) reported the same number of behaviors to minimize their cost of tobacco, and one person quit tobacco entirely, describing the tax as a motivating factor.

### 3.3. Factors Influencing Price-Minimizing Behaviors

Respondents reported that the tax increase made them think about quitting, reduce their tobacco use, or take specific actions to minimize the price of their cigarette purchases. There were five factors that emerged as impacting smokers’ efforts to save money on cigarettes after the tax: brand loyalty, frugality, addiction, stress, and acclimation.

#### 3.3.1. Brand Loyalty

In this study, we defined brand loyalty as consumers’ preference to continue using the same brand of cigarettes. Resistance to switching brands, even temporarily, was common, despite the tax increase and the money smokers might save by choosing a cheaper brand. As one participant shared, “Taste is taste, you can’t compromise … I choose taste, I guess, over price”. Others described the brand of the cigarette as a comfort, or even part of their identity, especially if they had a long history smoking that brand.

When brand switching did occur, it was most frequently for price, most commonly to take advantage of in-store promotions, or sometimes coupons mailed to their home. This seemed to be an easy way for participants to save a little money with minimal impact, because they usually switched to a brand at a similar price point and to cigarettes of a similar style.

Switching from packaged cigarettes to other tobacco such as cigarillos or pipe tobacco to make roll-your-own cigarettes resulted in a dramatically lower cost because pipe tobacco was taxed at a lower rate than loose cigarette tobacco at the time of the study. Many participants reported trying roll-your-own cigarettes (with either loose cigarette or pipe tobacco) at some time during their smoking history. However, most did not like loose tobacco because they disliked the taste, or as one interviewee stated: “(it) grosses me out”.

#### 3.3.2. Addiction

Of those who did make a permanent switch to using loose pipe tobacco in a roll-your-own cigarette, this change was a “last stop” for those most committed to smoking, and they tended to be the heaviest smokers in this sample (30 or more cigarettes a day). A subset of study participants referred to their addiction during the course of the interviews. Some acknowledged this as a reason why they smoked and as a particular barrier to their quitting. Others referred to their addiction more obliquely by describing price as unimportant because of how much they needed their cigarettes: “When you’re a smoker and you need (cigarettes) … you’ll pay whatever to get it”. Some only acknowledged their addiction after the tax increase, but not before. In this way, the tax helped them to see their addiction anew. The most popular metaphor (expressed by four participants) was that the need for cigarettes is like the need for gas to drive their cars, a non-negotiable need that a person pays for even if they do not like the price they have to pay for it.

#### 3.3.3. Stress

Stress appears to mediate price-minimizing behaviors, influencing participants to change their behaviors to accommodate stressful events. For example, one participant expressed the desire to quit smoking at Time 1 because he knew the tax was coming and the price of cigarettes was a very important motivation for quitting. At the Time 2 interview, he had reduced his consumption from eleven to eight cigarettes a day. He was also sharing fewer cigarettes and smoking half and saving half for later. Then he experienced a death in the family, which escalated his return to smoking half a pack a day and sharing his cigarettes again with friends and family. He appeared to have taken the energy that he spent on reduction and price minimizing and focused it instead on dealing with the stressful event.

In addition to isolated events, as described above, ongoing stress due to health, family, and other issues appeared to be part of a complex landscape of environmental and personal barriers against quitting for some participants. Stressful events seemed to both increase their need for cigarettes and decrease the energy they had available to reduce their cost of smoking, despite any difficulties faced in affording and accessing cigarettes. One man shared: “I was dealing with five people dying in my life … It brought out a lot of stress and made me smoke a lot more”. Many used price-minimizing behaviors to address the financial cost of buying cigarettes. Post-tax, these participants increased their efforts to preserve the role of cigarettes in helping them cope with their stress.

#### 3.3.4. Frugality

For this study, frugality is defined as having an expressed concern for the economy of one’s smoking and/or an interest in the “value” of cigarettes. This may be contrasted with participants who felt less pressure to save money on their purchases. Frugality appeared to be independent of income, with both higher- and lower-income individuals demonstrating the trait. A subset of participants seemed to possess a unique frugality that impacted their smoking and their efforts to save money on smoking in a variety of ways.

Frugality appeared associated with participants engaging in more price-minimizing behaviors, and some frugal study participants increased their efforts to save money after the tax. For example, one 39-year-old man living in a rural area with an annual household income of $60,000 and a household of one reported, “I’m trying to get myself addicted to these things (cheap cigars) for the price”, after the tax was implemented.

However, some smokers’ frugality was a source of conflict. Their desire to smoke conflicted in a painful way with their instinct to save money. For example, one smoker with an annual household income of more than $100,000 for a five-person household described herself as frugal and very price-conscious. She was angry at the government for taxing cigarettes because she believed it was unfair to target smokers for a tax and it was an intrusion in her life. She was also mad at herself for not being able to quit. Her frugality seemed to exacerbate her frustrations, and contributed additional pressure and conflict to her smoking.

#### 3.3.5. Acclimation and Resignation

A key mechanism by which participants maintained their smoking through the tax increase was acclimation. Before the tax increase, most participants seemed to have fairly stable and effective ways of affording cigarettes, even on limited incomes. A common first reaction to the tax increase for many was shock or dismay upon discovering the increased price of a pack of cigarettes (“sticker shock”), which was often accompanied by a desire to quit or reduce, or a commitment to switch to a lower-priced brand. However, the need for nicotine, the desire to smoke one’s preferred brand, and stress often conflicted with desires to smoke fewer cigarettes or quit. Many participants seemed to acclimate to the price increase so that the cost became the new and expected norm: “A lot of people rumble and talk about (the tax increase) … but I’m already starting to get used to it”. Others seemed to become dispirited or express feelings of resignation such as: “It is what it is”. Just before and after the tax, several participants reported receiving more coupons in the mail than usual from tobacco companies.

#### 3.3.6. Relationship of Factors

For a few participants the tax seemed to be governed primarily by a single factor. For example, a young man in rural Minnesota smoked 20 cigarettes a day and reported an annual household income of less than $20,000 for a household of four. During the study he became a father and caring for his child added stress. He switched brands to save money until he got a new job shortly after the tax was implemented. Then he continued to smoke his preferred brand. The financial freedom of a job impacted his smoking behavior more powerfully than the tax or the stress from being a new father.

Two or more factors seemed to co-exist with each other in complex and unique relationships for many more participants. For example, a female smoker with an annual household income of less than $10,000 for a household of two smoked 10 cigarettes a day and reported high levels of perceived stress. The financial impact of the tax was difficult for her, but she resisted moving to a cheaper brand because of taste. This conflicted with her sense of frugality, so she switched to the “100 s” style of her brand because she saw it as being a better value. Longer cigarettes unintentionally resulted in her smoking fewer cigarettes, and at Time 3 she reduced to smoking four cigarettes per day and expressed a strong commitment to quitting and described a plan for how she would achieve it.

Generally speaking, frugality tended to pair with reducing smoking and using price-minimizing behaviors. Being brand-loyal seemed to limit the price-minimizing options available, and brand loyalty and addiction appeared to coexist with acclimation to a higher price and smoking and buying behaviors consistent with baseline. Perceived stress existed together with increased smoking, thwarted reductions, and fewer price-minimizing behaviors (before and after the tax) for many. Participants’ frugality seemed to conflict with brand loyalty and addiction, sometimes generating greater perceived stress.

## 4. Discussion

Taken together, the results suggest that smokers face a complex array of pressures that shape their reactions to a substantial price increase. While many participants wished to quit tobacco, reduce their use, or switch brands in response to the new tax, many became acclimated to the price increase and maintained their smoking and cigarette-purchasing behaviors. Frugality appeared to support reductions in smoking and more price-minimizing behaviors both before and after the tax, but did conflict with participants’ brand loyalty and addiction. This dynamic sometimes resulted in increased stress and conflict over smoking. The relationship between addiction and price-minimizing behaviors has been suggested in other research [3,6], but remains an area requiring further investigation [19]. Similarly, White *et al.* [4] also suggest the role of brand loyalty in price-minimizing behaviors, but this is an area where more research is needed. In addition, this study’s examination of frugality is mirrored in a recent study of brand switching that confirms that longer cigarettes may indeed contain more nicotine, and that some smokers switch to them for better value [11].

Recent research suggests a change in patterns of smoking, whereby smokers in the United States and other countries are smoking less, and making more quit attempts [20]. The finding that a minority of participants reduced the number of cigarettes they smoked in order to continue smoking, as found in this study, complements Kulik and Glantz’s findings. Additionally, this study finds that other price-minimizing behaviors reported seem to fulfill the function of continued smoking.

Both reductions in cigarette use and price-minimizing behaviors were fluid after the tax. Some reductions and price minimizing were maintained from one month to three months post-tax, but many reverted to baseline levels. This represents a short-term impact of the tax increase, which is likely mediated by the inter-related factors influencing reduction and price minimizing. For example, reductions appear easily influenced by stress, addiction, and brand loyalty. Many smokers experience more than one of these conditions. These findings suggest that the period shortly before and immediately after a tax increase is very sensitive and turbulent for some smokers [21].

Therefore, immediate reductions in cigarette use post-tax should be seen as fragile, which is consistent with a small body of existing literature [22]. These findings support previous studies that report an increase in calls to telephone quitlines for a short period immediately before and after a tax, and that recommend timing cessation messages to capture quitters during this vulnerable time [21].

Based on these findings, a possible path forward is cessation-focused mass media that is carefully coordinated around a tax increase in order to encourage smokers to consider quitting before or after a tax increase. In addition messages should be developed to help smokers who struggle to maintain their new behaviors shortly after a tax increase. It may also be beneficial to tailor media messages to support a larger number of quits or reductions that smokers may have started absent a call to a cessation quitline. Recent research supporting messages to “cut down to quit” [23] may be particularly beneficial for those struggling to adopt and maintain reductions and quit attempts around the time of a tax increase.

The open-ended and formative nature of this study led to some insights about the construct of price-minimizing behaviors. Participants identified some actions they used to reduce their cigarette expenditures that are not commonly reported in the literature, such as sharing fewer cigarettes and smoking half and saving half for later. Additionally, short-term reductions often may be more accurately conceptualized as pric-minimizing behaviors as opposed to a desired tobacco user behavior given their fragility. This mirrors findings reported by the one other qualitative study found on price-minimizing behaviors [9]. Other researchers have examined the impact of tax increases and price-minimizing behaviors on reduced cigarette use [3,4]. In this study we found factors such as addiction, stress, and brand loyalty appear to encourage acclimation back to the original amount of cigarettes smoked per day in the short term. As the tobacco industry shifts its marketing to point of sale and direct to the consumer, tobacco control efforts will need to adjust with new policies designed to discourage marketing and promotion including bans on coupons, discounting, and other point-of-sale efforts [5].

Up to this point most literature on price-minimizing behaviors has used an econometric lens and has categorized a reduction in use as a desired outcome instead of as a set of complex behaviors. This study finds that smokers reduce their use of tobacco around a tax increase to either minimize the cost of smoking so they can continue to smoke, or as a pathway to quitting with the tax serving as a commitment device [3], although only one successful quit attempt was observed. Future studies may benefit from discriminating between these differing intentions for reduction.

## 5. Conclusions

In sum, this study suggests that tax increases are effective in promoting quit attempts, reducing cigarette consumption, and leading smokers to examine their smoking. This study contributes to the literature by exploring the context surrounding smokers’ decisions immediately before and after a price increase. Most study participants changed something about their smoking behavior after the tax, and they were doing so in a larger social environment that is increasingly hostile to their smoking. We found forces such as brand loyalty, frugality, addiction, and stress that conflict with each other and with a desire to reduce and quit. Some smokers become dispirited and resigned to their smoking and its financial toll.

Tobacco control advocates may be better able to assist these vulnerable smokers to quit, especially those with the lowest incomes, by carefully timing mass media messaging immediately before and after a tax increase and calibrating messaging to consider the contextual factors shown to be powerful in this study.

## Figures and Tables

**Table 1:** Interview content.

Time 1	Follow-Up
**Smoking Habits**	
Please walk me through when and where you smoke on a typical day? What is your smoking routine?	Are you still currently smoking cigarettes? I’d like to hear about your smoking in the last 30 days. Same items from Time 1. Has anything about your smoking routine changed since the last time we talked?
Do you prefer a certain brand? Why? How long have you smoked it? Have you ever switched? Why? When was that?	Are you still smoking (insert brand from last interview?). If YES: Have you thought at all about switching brands in the last 30 days? What if anything, would make you want to switch brands? If NO: What are you smoking now? Why did you switch? Why did you choose (new brand)?
How much of your cigarette do you smoke? All of it or part of it? When/why?	Same items in Time 1.
Do you currently smoke cigarettes every day, some days or not at all? If SOME DAYS: How many days do you smoke cigarettes in the last 30 days?	Same items in Time 1.
How many cigarettes do you smoke per day on the days that you smoke?	Same item in Time 1.
How soon after you wake up do you smoke your first cigarette?	Same item in Time 1.
**Buying Habits**	
Please walk me through the last time you bought cigarettes.	Same items in Time 1. Since we’ve talked last, has anything changed about what, when, or where you buy your cigarettes? If yes, what? Why?
Type of tobacco, brand and style.	
Pack or carton, number purchased.	
Where purchased.	
How much did you pay?	
Use of coupons or in-store promotions.	
How typical is this purchase? Is there anything about this purchase that is not typical?	Is this purchase typical of when you’ve bought cigarettes in the last 30 days? Is there anything about this purchase that is not typical?
How often do you typically buy cigarettes?	In the last 30 days, same item from Time 1.
Do you ever try new things or switch what you normally buy because of coupons or in-store specials? If Yes: what kinds of specials or deals spur you to switch what you buy?	In the last 30 days, have you tried any new things or switched what you normally buy because of coupons or in store specials? If Yes: what was it? What made you want to switch?
What kinds of coupons or in-store promotions do you ignore?	In the last 30 days, same item as Time 1.
**Price**	
	Since we’ve talked last would you say that you’ve paid more, less or about the same for your cigarettes? How much of an increase/decrease have you seen? Has it been the same everywhere?
How much do you think about price when you buy cigarettes?	Since we’ve talked last, (same item from Time 1).
How important is price to you in terms of what you buy? Where you buy?	Same item from Time 1.
Do you ever remember an occasion where you changed something about your smoking to save money? What is it? Tell me more about that. Prompt as necessary: What did you think of (that behavior)? How well did it work? Would you continue doing it/do it again?	Since we talked last, do remember an occasion where you changed something about your smoking to save money? What is it? Tell me more about that. Prompt as necessary: What did you think of (that behavior?). How well did it work? Would you continue doing it/do it again?
If you were to give advice to a friend about how to save money on tobacco, what advice would you give them? Have you ever done this? Why or why not?	Same item from Time 1.
	Time 3 only: you’ve talked about how (brand, convenience, taste) influence what you buy. Where does price fall when you consider all of these?
	Time 3 only: How easy is it for you to afford the cigarettes you are currently buying? Do you think you’d buy more cigarettes if you had more disposable income? Would you change your brand? Is it ever difficult for you to afford the cigarettes that you are currently buying? Have you ever not bought cigarettes because of finances? If Yes: how often?
**Quitting**	
In the last month, have you stopped smoking for 24 h or more because you were trying to quit? IF YES: When? For how long? Why did you want to quit? How did it go? IF NO: Have you used any other forms of tobacco besides cigarettes in the last 30 days? What types? (cigars/cigarillos/little cigars, pipe, chewing tobacco/snuff/dip/snus, other forms). For each type: How many days in the last 30? How much during the weeks that you used? Do you intend to quit in the next 30 days?	Same items as Time 1
In the last 30 days have you used an e-cigarette, also known as an electronic cigarette, at any time, even a puff? If YES: what made you decide to use them? How often do you use them? What did you think of it? The experience of smoking it? The nicotine rush? Do you plan to quit using e-cigarettes in the next 30 days?	Same items as Time 1
**Income (Time 3 Only)**	
	Is your annual household income from all sources … <$10,000, <$15,000, <$20,000, <$25,000, <$35,000, <$50,000, <$75,000, <$100,000.
	How many people live in your household?
**Health-Related Quality of Life (Time 3 Only)**	
	Would you say that in general, your health is excellent, very good, good, fair, or poor?
	Now thinking about your physical health, which includes physical illness and injury, for how many days during the past 30 days was your physical health not good?
	Now thinking about your mental health, which includes stress, depression, and problems with emotions, for how many days during the past 30 days was your mental health not good?
	During the past 30 days, for about how many days did poor physical or mental health keep you from doing your usual activities, such as self-care, work, or recreation?
**Perceived Stress (Time 3 only) Response Options: Never, Almost Never, Sometimes, Fairly Often, Very Often**	
	In the last month, how often have you felt that you were unable to control the important things in your life?
	In the last month, how often have you felt confident about your ability to handle your personal problems?
	In the last month, how often have you felt that things were going your way?
	In the last month, how often have you felt difficulties were piling up so high that you could not overcome them?

## References

[1] U.S. Department of Health and Human Services (2014). The Health Consequences of Smoking—50 Years of Progress.

[2] Chaloupka F.J., Yurekli A., Fong G.T. (2012). Tobacco taxes as a tobacco control strategy. Tob. Control.

[3] Choi K., Boyle R.G. (2013). Minnesota smokers’ perceived helpfulness of 2009 federal tobacco tax increase in assisting smoking cessation: A prospective cohort study. BMC Public Health.

[4] White V.M., Gilpin E.A., White M.M., Pierce J.P. (2005). How do smokers control their cigarette expenditures?. Nicotine Tob. Res..

[5] Xu X., Wang X., Caraballo R.S. (2016). Is every smoker interested in price promotions? An evaluation of price-related discounts by cigarette brands. J. Public Health Manag. Pract..

[6] Dunlop S.M., Perez D., Cotter T. (2011). Australian smokers’ and recent quitters’ responses to the increasing price of cigarettes in the context of a tobacco tax increase. Addiction.

[7] Licht A.S., Hyland A.J., O’Connor R.J., Chaloupka F.J., Borland R., Fong G.T., Nargis N., Cummings K.M. (2011). How do price minimizing behaviors impact smoking cessation? Findings from the International Tobacco Control (ITC) Four Country Survey. Int. J. Environ. Res. Public Health.

[8] Pesko M.F., Kruger J., Hyland A. (2012). Cigarette price minimization strategies used by adults. Am. J. Public Health.

[9] Guillaumier A., Bonevski B., Paul C. (2015). “Cigarettes are priority”: A qualitative study of how Australian socioeconomically disadvantaged smokers respond to rising cigarette prices. Health Educ. Res..

[10] Choi K., Hennrikus D., Forster J., St Claire A.W. (2012). Use of price-minimizing strategies by smokers and their effects on subsequent smoking behaviors. Nicotine Tob. Res..

[11] Cornelius M.E., Cummings K.M., Fong G.T., Hyland A., Driezen P., Chaloupka F.J., Hammond D., O’Connor R.J., Bansal-Travers M. (2015). The prevalence of brand switching among adult smokers in the USA, 2006–2011: Findings from the ITC U.S. surveys. Tob. Control.

[12] Xu X., Malarcher A., O’Halloran A., Kruger J. (2014). Does every U.S. smoker bear the same cigarette tax?. Addiction.

[13] Xu X., Pesko M.F., Tynan M.A., Gerzoff R.B., Malarcher A.M., Pechacek T.F. (2013). Cigarette price-minimization strategies by U.S. smokers. Am. J. Prev. Med..

[14] Cornelius M.E., Driezen P., Fong G.T., Chaloupka F.J., Hyland A., Bansal-Travers M., Carpenter M.J., Cummings K.M. (2014). Trends in the use of premium and discount cigarette brands: Findings from the ITC U.S. Surveys (2002–2011). Tob. Control.

[15] Amato M.S., Boyle R.G., Brock B. (2015). Higher price, fewer packs: Evaluating a tobacco tax increase with cigarette sales data. Am. J. Public Health.

[16] Miles M.B., Huberman A.M., Saldana J. (2014). Qualitative Data Analysis: A Method Sourcebook.

[17] Saldana J.M. (2003). Longitudinal Qualititative Research: Analyzing Change Through Time.

[18] Sandelowski M. (2000). Whatever happened to qualitative description?. Res. Nurs. Health.

[19] Bader P., Boisclair D., Ferrence R. (2011). Effects of tobacco taxation and pricing on smoking behavior in high risk populations: A knowledge synthesis. Int. J. Environ. Res. Public Health.

[20] Kulik M.C., Glantz S.A. (2015). The smoking population in the USA and EU is softening not hardening. Tob. Control.

[21] Keller P.A., Greenseid L.O., Christenson M., Boyle R.G., Schillo B.A. (2015). Seizing an opportunity: Increasing use of cessation services following a tobacco tax increase. BMC Public Health.

[22] Betzner A.E., Boyle R.G., Luxenberg M.G., Schillo B.A., Keller P.A., Rainey J., Capesius T., Saul J.E. (2012). Experience of smokers and recent quitters with smoking and quitting. Am. J. Prev. Med..

[23] Begh R., Lindson-Hawley N., Aveyard P. (2015). Does reduced smoking if you can’t stop make any difference?. BMC Med..

